# Fibulin-1 is epigenetically down-regulated and related with bladder cancer recurrence

**DOI:** 10.1186/1471-2407-14-677

**Published:** 2014-09-18

**Authors:** Wei Xiao, Ji Wang, Heng Li, Ding Xia, Gan Yu, Weimin Yao, Yang Yang, Haibing Xiao, Bin Lang, Xin Ma, Xiaolin Guo, Wei Guan, Hua Xu, Jihong Liu, Xu Zhang, Zhangqun Ye

**Affiliations:** Department of Urology, Tongji Hospital, Tongji Medical College, Huazhong University of Science and Technology, Wuhan, 430030 China; Translational Medicine Center, Tongji Hospital, Tongji Medical College, Huazhong University of Science and Technology, Wuhan, 430030 China; Department of Urology and Helen-Diller Comprehensive Cancer Center, University of California, San Francisco, California USA; Department of Urology, PLA General Hospital, Military Postgraduate Medical College, Beijing, 100853 China; School of Health Sciences, Macao Polytechnic Institute, Macao, China

**Keywords:** Fibulin-1, Bladder cancer, Promoter hypermethylation, Tumor suppressor gene, Cancer recurrence

## Abstract

**Background:**

Bladder cancer is one of the most common cancers worldwide. Fibulin-1, a multi-functional extracellular matrix protein, has been demonstrated to be involved in many kinds of cancers, while its function in bladder cancer remains unclear. So here we investigated the expression and function of fibulin-1 in Bladder cancer.

**Methods:**

We used real-time PCR, Western blot analysis and immunohistochemistry to determine the expression of fibulin-1 in Bladder cancer cells and patient tissues respectively. Methylation-specific PCR and quantitative sequencing were used to examine the methylation status of FBLN1 gene promoter. Eukaryotic expression plasmid and lentiviral vector were used to overexpress fibulin-1 in Bladder cancer cells 5637, HT-1376 to investigate its function *in vitro* and *in vivo*.

**Results:**

We identified that fibulin-1 was significantly down-regulated in bladder cancer, and its dysregulation was associated with non-muscle-invasive bladder cancer (NMIBC) grade and recurrence. The promoter region of FBLN1 was generally methylated in bladder cancer cell lines and tissues, further investigation in patient tissues showed that the methylation status was associated with the fibulin-1 expression. Overexpression of fibulin-1 significantly suppressed tumor growth, induced tumor cell apoptosis, decreased cell motility, and inhibited angiogenesis in cultured bladder cancer cells and xenograft tumor in nude mice.

**Conclusions:**

Altogether, our results indicated that fibulin-1 expression is associated with NMIBC grade and recurrence, it is epigenetically down-regulated and functions as a tumor suppressor gene and angiogenesis inhibitor in bladder cancer.

**Electronic supplementary material:**

The online version of this article (doi:10.1186/1471-2407-14-677) contains supplementary material, which is available to authorized users.

## Background

Bladder cancer is the fifth most common malignant disease in the Western world [1]. Just in US, 70,530 new patients and 14,680 deaths recorded in 2010 [2]. Approximately 75% of patients with BC present with a disease that is confined to the mucosa (stage Ta, CIS) or submucosa (stage T1). These categories are grouped as non-muscle-invasive bladder tumors. Non-muscle invasive BC (NMIBC) has a high prevalence due to low progression rates and long-term survival in many cases while it represents a heterogeneous group of tumors with different rates of recurrence, progression, and disease-related mortality [3]. In an effort to improve current diagnosis and management of bladder cancer, intense researches on identifying clinically helpful tumor markers or potentially valuable therapeutic targets have been carried out worldwide [4].

Despite the environment factors such as smoking and occupational exposures, it is now widely accepted that genetic and epigenetic alterations of genome are associated with bladder cancer risk. Genome-wide association studies of bladder cancer identified single-nucleotide polymorphisms (SNPs) on chromosome 8q24, upstream of the MYC oncogene, on chromosome 3q28 near the TP63 tumor suppressor gene [5], and in the PSCA gene to be associated with bladder cancer risk [6]. DNA methylation is one of the most consistent epigenetic changes occurring in human cancers [7, 8]. And it is well established that aberrant hypermethylation of the promoter region of tumor suppressor genes is associated with transcriptional silencing, and that hypermethylation is an alternative mechanism of functional inactivation [9] Moreover, promoter hypermethylation of tumor-related gene has also been proposed as a novel biomarker for detecting cancer and predicting prognosis [10].

Recent evidence increasingly points to the important role of stromal extracellular matrix (ECM) components in tumor progression; many of ECM proteins interact directly with tumor cells, via integrins and other cell-surface receptors, to influence functions such as proliferation, apoptosis, migration and differentiation [11]. Fibulin-1 belongs to a growing family of extracellular glycoprotein. Functionally, fibulin-1 binds to many extracellular matrix (ECM) proteins, including laminin, fibrinogen, fibronectin, nidogen-1, and endostatin, and to the proteoglycans, aggrecan and versican [12, 13]. Mice lacking fibulin-1 die perinatally and display vascular anomalies in the kidney in addition to extensive hemorrhage in several organs, likely related to abnormalities in endothelial cell interactions with subendothelial ECM [14]. Fibulin-1 is reported to involve in the progression of many kinds of cancers, such as breast, ovarian and prostate cancer [15–18]. Besides, fibulin-1 has been identified epigenetically silenced in gastric cancer and hepatocellular carcinoma through promoter hypermethylation [19, 20]. However, the association between fibulin-1 and bladder cancer remains unknown. Here we report that fibulin-1 expression is associated with NMIBC grade and recurrence, it is epigenetically down-regulated and functions as a tumor suppressor gene and angiogenesis inhibitor in bladder cancer.

## Methods

### Patient and sample collection

The cohort included 139 consecutive patients with NIMBC treated by TURBT in Chinese People’s Liberation Army General Hospital from December 2008 to September 2009. All tumors were initially staged, graded and classified by pathologists with expertise in genitourinary pathology according to 2002 TNM classification and 2004 WHO grading system. Adjuvant therapy of NIMBC after TURBT included intravesical instillation and chemotherapy. Follow-up information obtained from medical records of the patients who fulfilled inclusion criteria included tumor stage, grade, the development of tumor recurrence, the presence of multifocal versus unifocal tumor growth, the co-existence of CIS as well as patient gender and age. Briefly, patients were seen postoperatively at least every 3 to 4 months for the first 2 years and semiannually thereafter. Besides that, telephone follow up was taken every month. Recurrence was defined as a new tumor appearing in the bladder after initial clearance. Recurrence free survival (RFS) was calculated as the time from TURBT to the date of the first documented bladder tumor recurrence. The mean follow-up period for the study was 38 months (range, 33–43 months). There was no case of death in the study. The study protocol was approved by the Institutional Ethics Committee of Huazhong University of Science and Technology, Tongji Hospital and Chinese People’s Liberation Army General Hospital, and a written informed consent was obtained from all participants involved in the study.

### Cell culture and transfection

Muscle-invasive bladder cancer cell lines 5637, T24, J82 and HT1376 were purchased from ATCC and maintained in RPMI-1640 medium supplemented with 10% fetal bovine serum (FBS) in a humidified atmosphere of 5% CO_2_ maintained at 37°C. SV-HUC-1 was also purchased from ATCC and maintained in DMEM/F-12 medium. Primary human umbilical vein endothelial cell (HUVEC) and Endothelial Cell Medium were purchased from ALLCELLS, LLC (Shanghai, China). Plasmid transfection was carried out using FuGene HD Transfection Reagent (Roche), according to the manufacture’s protocol.

### Plasmid, lentivirus and infection

Generally, the full-length coding sequence (CDS) of FBLN1 was amplified from pBluescript-FBLN1 (Thermo scientific) and constructed into eukaryotic expression vector pEGFP-N1 (Clontech) or lentivirus clone vector pCDH-CMV. Then the lentivirus was packaged and purified according to the manufacture’s protocol (SBI, USA). The MOI for 5637 and HT1376 cells was 10 and 5 respectively.

### Immunohistochemical (IHC) staining

Besides the specimens of 139 patients in follow-up cohort, 17 normal or adjacent normal bladder tissue specimens conserved in urology institution, Tongji hospital were also included in this study. Methods for immunohistochemical (IHC) staining tissue slides have been described previously [21]. Depending on the percentage of positive cells and staining intensity, fibulin-1 staining was classified into three groups: negative, weak positive and strong positive. Specifically, the percentage of positive cells was divided into five grades (percentage scores): <10% (0), 10–25% (1), 25–50% (2), 50–75% (3), and 75% (4). The intensity of staining was divided into four grades (intensity scores): no staining (0), light brown (1), brown (2), and dark brown (3). Fibulin-1 staining positivity was determined by the formula: overall scores = percentage score × intensity score. The overall score of ≤3 was defined as negative, of >3 and ≤6 as weak positive, and of >6 as strong positive. In some analysis, weak positive and strong positive were combined as positive to suit the paired statistical analysis.

### Western blot analysis

Cells were prepared by washing with PBS. Protein extraction and western blot analysis were performed as previously described [22]. Primary antibodies included fibulin-1 (1:500 dilutions; Abcam) and GAPDH (1:1000 dilution; Cell Signaling Technology).

### mRNA expression analysis

Total RNA was isolated using RNeasy Mini Kit (Qiagen) and reverse transcribed using PrimeScript RT Master Mix (TaKaRa). The resulting cDNA samples were amplified by real-time PCR using gene-specific primer sets in conjunction with SYBR Premix Ex Taq (TaKaRa). All the primer sequences were listed in Additional file 1: Table S1.

### DNA methylation analysis by pyrosequencing and methylation specific PCR

Genomic DNA was isolated using QIAamp DNA Mini Kit (Qiagen) and bisulfite modification of the genomic DNA was carried out using an Epitect Bisulfite Kit (Qiagen) according to the manufacturer’s instructions. Methylation Specific PCR (MSP) primers were designed with Methprimer (http://epidesigner.com). Pyrosequencing for five CpG sites in the promoter region was performed on a PyroMark Q96 instrument (Qiagen) according to manufacturer’s protocol, as described previously [23]. Record was analyzed by the manufacturer’s software. All the primer sequences were listed in Additional file 1: Table S1.

### Cell proliferation assay

5637 and HT1376 cells were infected with Lenti-NC or Lenti-FBLN1 for 48 h. Following treatments, cells were transferred to 96-well microplates and seeded at a density of approximately 800 cells per well. Fetal bovine serum proportion in the culture medium was decreased to 5% to avoid overgrowth in 72 h. Cell viability was subsequently determined every 24 h for three days by using the Cell Counting Kit-8 (CCK-8, Dojindo) according to the manufacturer’s protocol and Microplate reader (Thermo) to measure the absorbance.

The effect of fibulin-1 suppression on proliferation was also tested by the EdU incorporation assay. Briefly, 5637 and HT1376 cells were infected with Lenti-NC or Lenti-FBLN1 for 48 h. Following treatments, cells were transferred to 96-well microplates and seeded at a density of approximately 3 × 10^3^ cells per well for 12 h. Then, cells were incubated with 50 nM of EdU for an additional 2 h at 37°C. Cells were fixed with 4% formaldehyde for 15 min at room temperature and treated with 0.5% Triton X-100 for 20 min at room temperature to permeabilize cells. After being washed with PBS three times, cells were incubated with 1× Apollo reaction cocktail (100 μl/well) for 30 min. DNA was stained with 10 μg/ml of Hoechst 33342 stain (100 μl/well) for 20 min and visualized with fluorescence microscopy. Five groups of confluent cells were randomly selected from each sample image. EdU-positive cells were obtained from the fluorescent image, and the relative positive ratio was calculated from the average of the five group values.

### Colony formation assay

Exponentially growing cells were seeded at approximately 1,000 cells per well in 6-well plates after infection with Lenti-NC or Lenti-FBLN1. Culture medium was changed every three days. Colony formation was analyzed ten days following infection by staining cells with 0.05% crystal violet solution for 20 min.

### Apoptosis analysis

Flow cytometry for cell-apoptosis analysis was performed as previously described [24]. Briefly, 5637 and HT1376 cells were transfected with pEGFP-N1, pEGFP-FBLN1 or mock for 72 h. Then cells were collected and stained with Annexin V-PE and 7-AAD. The early stage apoptosis cells were detected for Annexin V-PE^+^/7-AAD^−^.

### *In vitro*migration and invasion assay

5637 and HT1376 cells were transfected with pEGFP-N1 or pEGFP-FBLN1 for 72 h. About 1 × 10^5^ of 5637 cells or 6 × 10^4^ HT1376 cells were plated in the upper chambers of 24-well Transwell plates (Corning) in FBS-free medium. Complete medium (10% FBS) was deposited in the lower chambers to serve as a chemo-attractant. After 12 h for 5637 or 10 h for HT1376, cells remaining on the upper filter were removed, while cells that passed through the Transwell filter were stained by 0.5% crystal violet solution for 15 min. Images were taken of six random optical fields (200×) on each filter and cell number was quantified by utilizing the Image-Pro Plus analysis software (Media Cybernetics). To evaluate cell invasion, Transwell membranes were coated with Matrigel (BD Biosciences) prior to plating infected cells. The Matrigel served as a basement membrane barrier that cells would have to destroy in order to invade the lower chamber. After 22 h for 5637 or 18 h for HT1376, crystal violet staining and cell counting were performed as above.

### *In vitro*tube formation assay

HUVECs were maintained in basic medium containing 2% FBS and 1% penicillin/ streptomycin or the indicated conditioned media (CM) of 5637 cells. HUVECs (3 × 10^4^) were seeded into a 96-well culture plate precoated with Matrigel (BD Biosciences) overnight and then cultured in the indicated condition. After 24 h incubation, the formation of tubes was photographed with a phase contrast microscopy (10× magnification, Olympus Instruments, Inc.), and quantified by counting branch points in five randomly selected microscope fields per well. The experiments were conducted twice in duplicate.

### Animal experiments

Tumorgenesis in nude mice was determined as described previously [25]. Generally, 5637 bladder cancer cells were infected with Lenti-NC and Lenti-FBLN1 respectively, 72 h after infection, cells were treated and passaged with medium containing 2 μg/ml puromycin to establish stable fibulin-1–expressing cell lines, and then cells were injected into the right flank of 6-week-old nude mice. Two groups of five mice each were injected subcutaneously with prepared cells at a single site. Tumor onset measured with calipers at the site of injection weekly by two trained laboratory staffs at different times on the same day. Tumor volume was calculated using the formula, 0.5ab^2^, where a represent the larger and b represents the smaller of the two perpendicular indexes. Animals were sacrificed 28 days after injection. These tumors were weighed and verified by hematoxylin and eosin (H&E) staining. The *in vivo* apoptosis was evaluated by TUNEL, and the vascularity evaluation was taken by immunohistochemical staining with CD31 antibody (Abcam). The study was conducted after getting approval from the Institutional Experimental Animal Ethical Committee, Huazhong University of Science and Technology, China.

### Statistical analysis

Statistical significance was determined by using the SPSS 15.0. The Fisher’s exact test was utilized to assess the significance between different proportions. Analysis of continuous variables between different groups was assessed by t test. Values are expressed as mean ± SEM unless otherwise indicated. RFS (recurrence-free survival) curves were constructed using the Kaplan-Meier method, and were compared with the log-lank test. The Cox proportional hazards model was used to assess the prognostic indicators for recurrence. The risk ratio and its 95% confidence interval were recorded for each marker. All statistical tests were two-sided, and significance was defined as p<0.05.

## Result

### Down-regulation of fibulin-1 expression levels in primary bladder cancer

**Figure 1:**
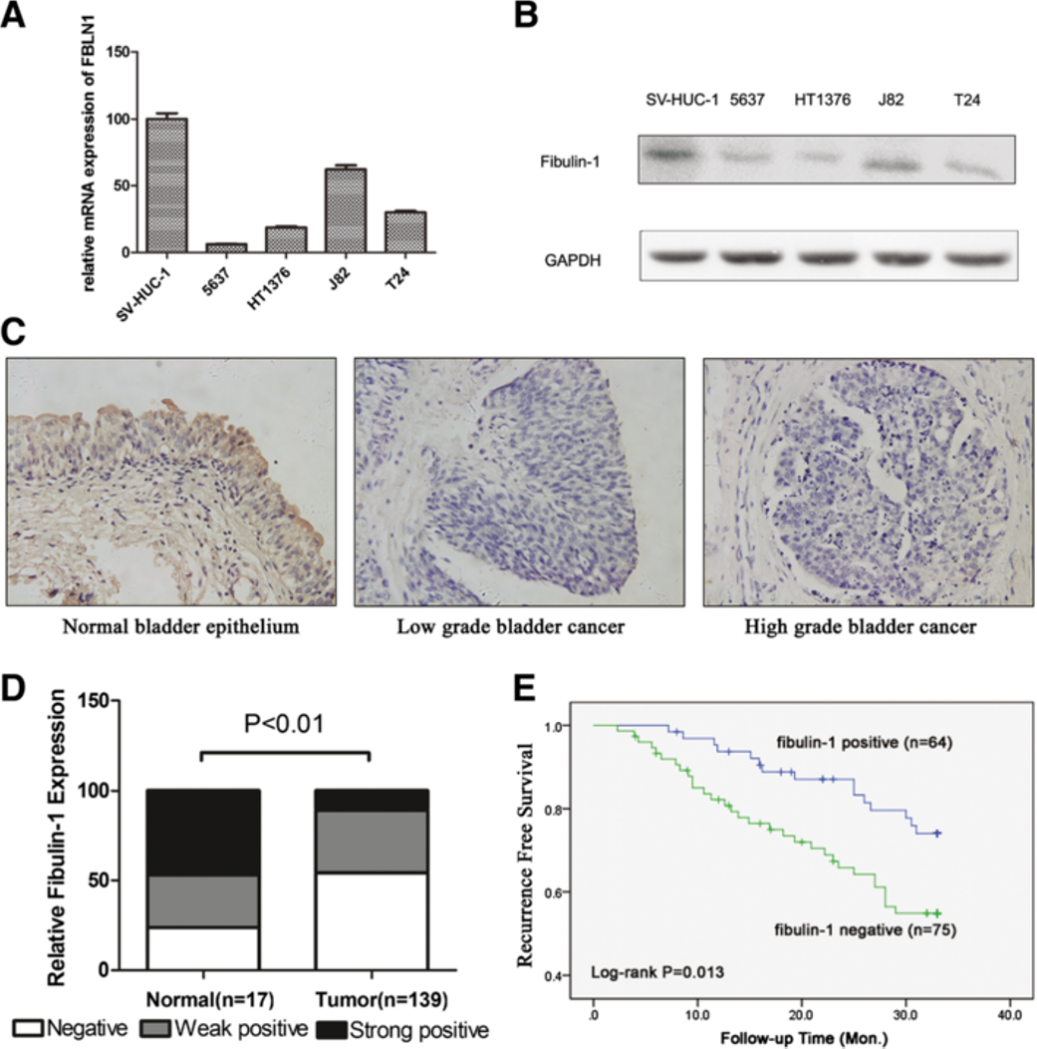
**Fibulin-1 was down-regulated in bladder cancer. A)** Fibulin-1 mRNA and **B)** protein expression levels were evaluated by qPCR and Western blot assay respectively in bladder cancer cell lines. GAPDH served as an internal control and loading control. **C)** Representative microphotographs of fibulin-1 staining in patient tissues. **D)** Summary of fibulin-1 expression determined by immunohistochemistry in NMIBC patient tissue samples. **E)** Kaplan-Meier estimate of recurrence-free survival stratified by fibulin-1 expression in 139 NMIBC patients.

Firstly, the expression levels of fibulin-1 in 4 bladder cancer cell lines (5637, HT1376, J82 and T24) and a non-tumorigenic bladder cell line SV-HUC-1 were evaluated by qPCR and Western blot respectively. Compared to SV-HUC-1 cells, all of bladder cancer cell lines had a significantly lower level of fibulin-1 expression in both mRNA (Figure 1A) and protein levels (Figure 1B).

Fibulin-1 expression was further analyzed by immunohistochemistry in a tissue microarray containing 139 non-muscle invasive bladder cancer and 17 normal or adjacent normal bladder tissue specimens (Figure 1C). A highly significant (Figure 1D, P < 0.01) difference between the normal and tumor tissues was observed as follows: 23.5% (4 of 17) negative, 29.4% (5 of 17) weak positive and 47.1% (8 of 17) strong positive staining of fibulin-1 in normal bladder specimens, whereas 54.0% (75 of 139) negative, 35.2% (49 of 139) weak positive and 10.8% (15 of 139) strong positive staining of fibulin-1 in bladder cancer samples. Importantly, loss of fibulin-1 expression was associated with tumor grade (Table 1, P < 0.05), but not with other clinicopathological parameters such as age, sex or tumor stage (Table 1, P > 0.05).

**Table 1:** **Clinicopathological features of fibulin-1 expression in 139 NMIBC patients**

Characteristic	Total (n = 139)	Fibulin-1 expression			P-value
		Negative (75)	Weak (49)	Strong (15)	
Sex					0.909
Men	102	54 (52.9%)	37 (36.3%)	11 (10.8%)	
Women	37	21 (56.8%)	12 (32.4%)	4 (10.8%)	
Age (years)					0.657
<average(62)	64	32 (50.0%)	24 (37.5%)	8 (12.5%)	
≥average(62)	75	43 (57.3%)	25 (33.3%)	7 (9.4%)	
Grade					<0.01*
Low grade	91	40 (44.0%)	37 (40.7%)	14 (15.4%)	
High grade	48	35 (72.9%)	12 (25.0%)	1 (2.1%)	
Stage					0.225
P_Ta_	63	36 (57.1%)	18 (28.6%)	9 (14.3%)	
P_T1_	76	39 (51.3%)	31 (40.8%)	6 (7.9%)	

*Two-tailed fisher’s exact test was done to determine the relationship of fibulin-1 expression with various variables and statistical significance was set at P < 0.05.

### Analysis of the association between NMIBC recurrence and clinicopathological parameters

We then analyzed recurrence-free survival rates to assess the prognostic significance of the expression of fibulin-1. The overall recurrence-free survival (RFS) rate of the 139 NMIBC patients was 66.9%. When assessed by Kaplan–Meier curves, patients with negative fibulin-1 expression tended to have significantly poorer RFS rates than those in the positive fibulin-1 expression group; 58.7% (negative fibulin-1 expression) and 76.6% (positive fibulin-1 expression) respectively (Figure 1E, log-rank test, P = 0.013). When we evaluated whether fibulin-1 negative expression was independently associated with RFS, several factors were subsequently investigated in COX regression analysis. As shown in Table 2, fibulin-1 negative expression was a significant prognostic factor in COX regression analysis for RFS (RR: 2.102, 95% CI: 1.130-3.912, P = 0.019).

**Table 2:** **Multivariate Cox regression analysis of potential risk factors for early recurrence of NMIBC**

Characteristic	P (cox-regression)	Risk Ratio (RR)	95% confidence interval	
			Lower	Upper
Sex	0.548	0.786	0.409	1.510
Age	0.225	0.754	0.419	1.356
Grade	0.000^*^	6.326	3.318	11.392
Stage	0.512	1.085	0.597	1.974
Fibulin-1 expression	0.019^*^	2.102	1.130	3.912

*P value was considered to indicate statistical significance.

### Promoter methylation analysis of fibulin-1 in bladder cancer

**Figure 2:**
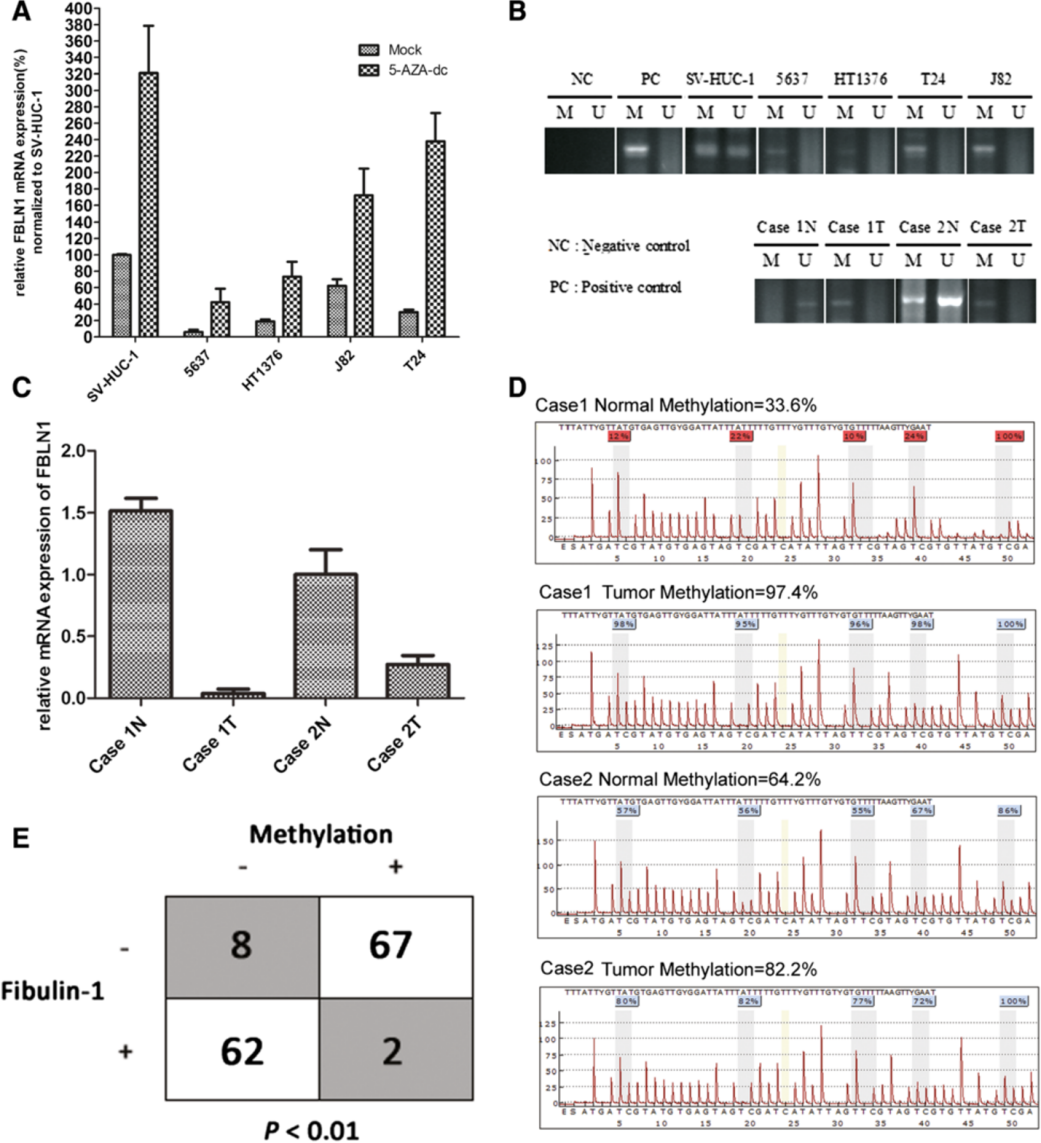
**Fibulin-1 was silenced in bladder cancer by promoter hypermethylation. A)** fibulin-1 expression in five bladder cell lines with or without 5-aza-dC treatment was determined by real-time RT-PCR. The results are the average of three independent experiments normalized to GAPDH levels. **B)** fibulin-1 methylation status in 5 bladder cell lines and two matched pairs of normal (N)/tumor (T) bladder tissues were analyzed by MSP. (M) amplification using primers specific for methylated DNA. (U) amplification using primers specific for unmethylated DNA. IVD (*in vitro* methylated DNA) and ddH_2_O were positive and negative controls, respectively. **C)** qPCR was used to analyze fibulin-1 expression in two matched pairs of bladder tissues mentioned in **B)**. **D)** Pyrosequencing of fibulin-1 promoter region two matched pairs of bladder tissues mentioned in **B)**, the average methylation rate of 5 CpG was listed. **E)** The correlation analysis of fibulin-1 expression and methylation in 139 NMIBC patient samples.

A typical CpG island (CGI) was found around fibulin-1 promoter using the MethPrimer (http://www.urogene.org/methprimer/index1.html). To explore whether promoter hypermethylation leads to the suppression of expression, we examined the expression of FBLN1 in bladder epithelial cell lines treated with the DNA methylation inhibitor, 5-aza-dC. After treatment, all the five cell lines showed a reactivation of FBLN1 expression (Figure 2A). To further detect the promoter methylation status of the fibulin-1 qualitatively and quantitatively, the promoter CpG islands in bladder epithelial cell lines and bladder epithelial tissue was determined by MSP and quantitative sequencing. As shown in Figure 2B, MSP results showed hypermethylation was detected in all bladder cancer cell lines, while partial methylation in non-tumorigenic cell line SV-HUC-1, which complied with the 5-aza-dC detection. For tissues, all two bladder cancer tissues showed hypermethylation while cancer adjacent tissues showed partial methylation or unmethylation, which complied with the mRNA analysis (Figure 2C). To ascertain MSP reliability, pyrosequencing of the two paired tissues were performed, and the results generally agreed with MSP results (Figure 2D). Then we systematically analyzed the MSP and IHC results of the 139 patient tissue samples. Remarkably, a statistically significant (P < 0.01, two-tailed χ^2^ test) inverse correlation between fibulin-1 expression and methylation was found (Figure 2E). Together, these results suggested that fibulin-1 was down-regulated through promoter hypermethylation in bladder cancer.

### Fibulin-1 suppressed bladder cancer cells proliferation and tumorigenicity

**Figure 3:**
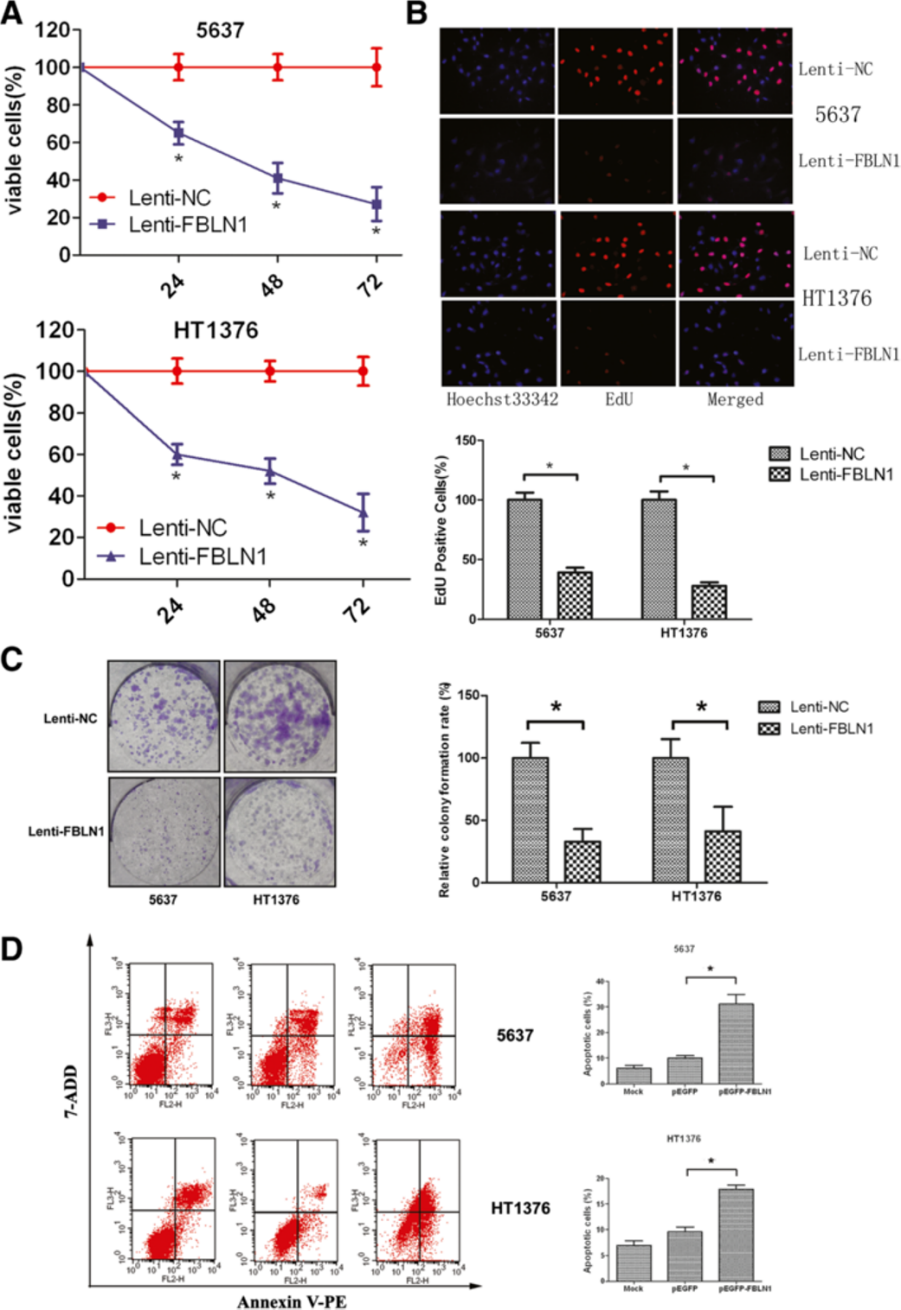
**Fibulin-1 functioned as a tumor suppressor in bladder cancer cells.** The effect of ectopic FBLN1 expression on tumor cell proliferation was investigated by **A)** CCK-8 and **B)** EdU assay in 5637 and HT1376 cells. Data are plotted as the mean ± SD of 3 independent experiments relative to mock treatments. **C)** The effect of ectopic FBLN1 expression on tumor cell tumorgenesis was investigated by the monolayer colony-formation assay. Quantitative analyses of colony numbers are shown as mean ± SD. **D)** Fibulin-1 induced apoptosis of bladder cancer cells. The early stage apoptosis cells were detected for Annexin V-PE+/7-AAD-. Quantitative analyses of apoptotic cell numbers are shown in the right panel as values of mean ± SD. Asterisk indicates p < 0.05.

Current data indicate that FBLN1 might have a tumor suppressive function. We investigated its tumor suppressive function by a gain-of-function strategy. We first detected fibulin-1’s function on cell proliferation by CCK-8 and EdU assay. After re-expression of FBLN1 in 5637 and HT1376 cells, the number of viable cells significantly reduced compared to the negative control (Figure 3A, P < 0.05). The EdU detection confirmed that re-expression of fibulin-1 could effectively inhibit cancer cell proliferation (Figure 3B, P < 0.05). Then, the fibulin-1’s function on tumorigenicity was detected *in vitro.* A lentivirus carrying FBLN1 was used in this study. After infected with Lenti-NC or Lenti-FBLN1 respectively, a monolayer colony formation assay was carried out, the results showed that the colonies formed on the plate were significantly decreased (Figure 3C, P < 0.01). These results suggested that fibulin-1 suppressed bladder cancer cells proliferation and tumorigenicity *in vitro*.

### Fibulin-1 induced bladder cancer cells apoptosis

Next, apoptosis in cancer cells were detected. For bladder cancer cells 5637 and HT1376, we used flow cytometry analysis based on Annexin V staining, as shown in Figure 3D, 72 h post transfection, the average early apoptosis rates (Annexin V-PE^+^/7-AAD^−^) of FBLN1-transfected 5637 and HT1376 cells were 31.12% and 17.91% respectively, which were significantly higher when compared to the control vector-transfected cells (10.04% and 9.64%, P < 0.05).

### Fibulin-1 suppressed bladder cancer cells motility and angiogenesis

**Figure 4:**
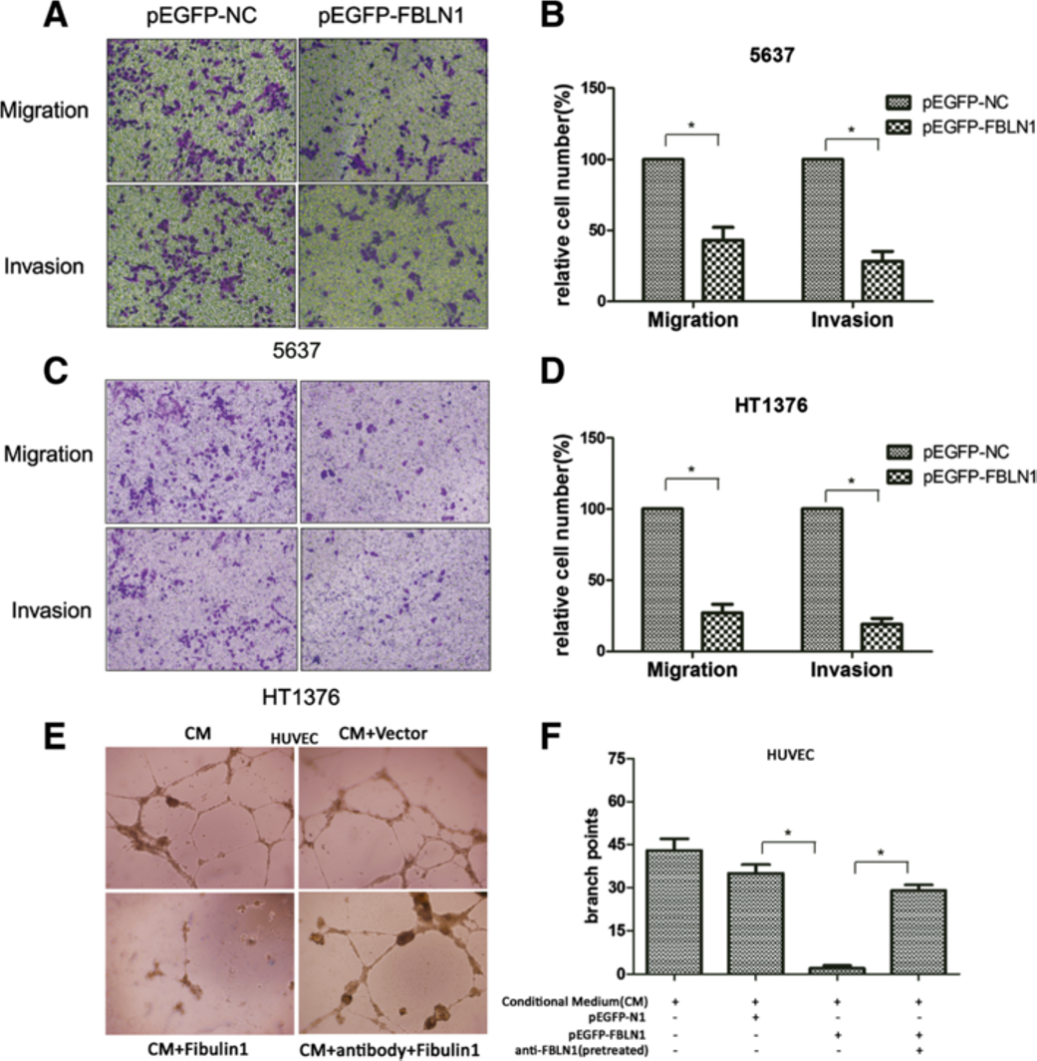
**Fibulin-1 inhibited motility and angiogenesis activation of bladder cancer cells. A)** 5637 and **C)** HT1376 cells were transfected with pEGFP-N1 or pEGFP-FBLN1 for 72 h and then the abilities of cell migration and invasion were detected respectively. Representative images showed results of one assay, **B)** and **D)** the column chart showed the mean ± SD of five randomly selected microscope fields per well. **E)** Tube formation of HUVECs was determined by assaying the numbers of branch nodes after 24 h of culture under a phase contrast microscope. HUVECs were cultured in the following media: conditioned media (CM) of 5637 cells, CM of 5637 cells transfected with pEGFP-N1, CM of 5637 cells transfected with pEGFP-FBLN1 and CM of 5637 cells transfected with pEGFP-FBLN1 with fibulin-1 antibody pretreatment. **F)** The summary and statically analysis of **E)**. All these experiments were repeated at least three times. Asterisk indicates p < 0.05.

As a novel ECM protein, we hypothesized that fibulin-1 may also play a role in bladder cell motility. To test this hypothesis, 5637 and HT1376 bladder cancer cells, which contain promoter methylation and lack fibulin-1 expression, were transfected to overexpress fibulin-1. Analysis of cell migration and invasion by Transwell assays revealed that fibulin-1 expression significantly suppressed migration and invasion of 5637 (Figure 4A and B) and HT1376 (Figure 4C and D) cell lines (P < 0.05).

To characterize the *in vitro* effects of fibulin-1 on angiogenesis, an endothelial tube assay was performed. Tube formation by activated HUVECs was achieved by the conditioned media (CM) of 5637 cells or CM of 5637 cells transfected with pEGFP-N1 vehicle. The angiogenic activity of CM lost by ectopic overexpression of fibulin-1, while it was restored by pretreated with antibody against fibulin-1 (Figure 4E and F). The results of this assay demonstrated that fibulin-1 inhibited angiogenesis *in vitro*.

### *In vivo*tumor study with fibulin-1

**Figure 5:**
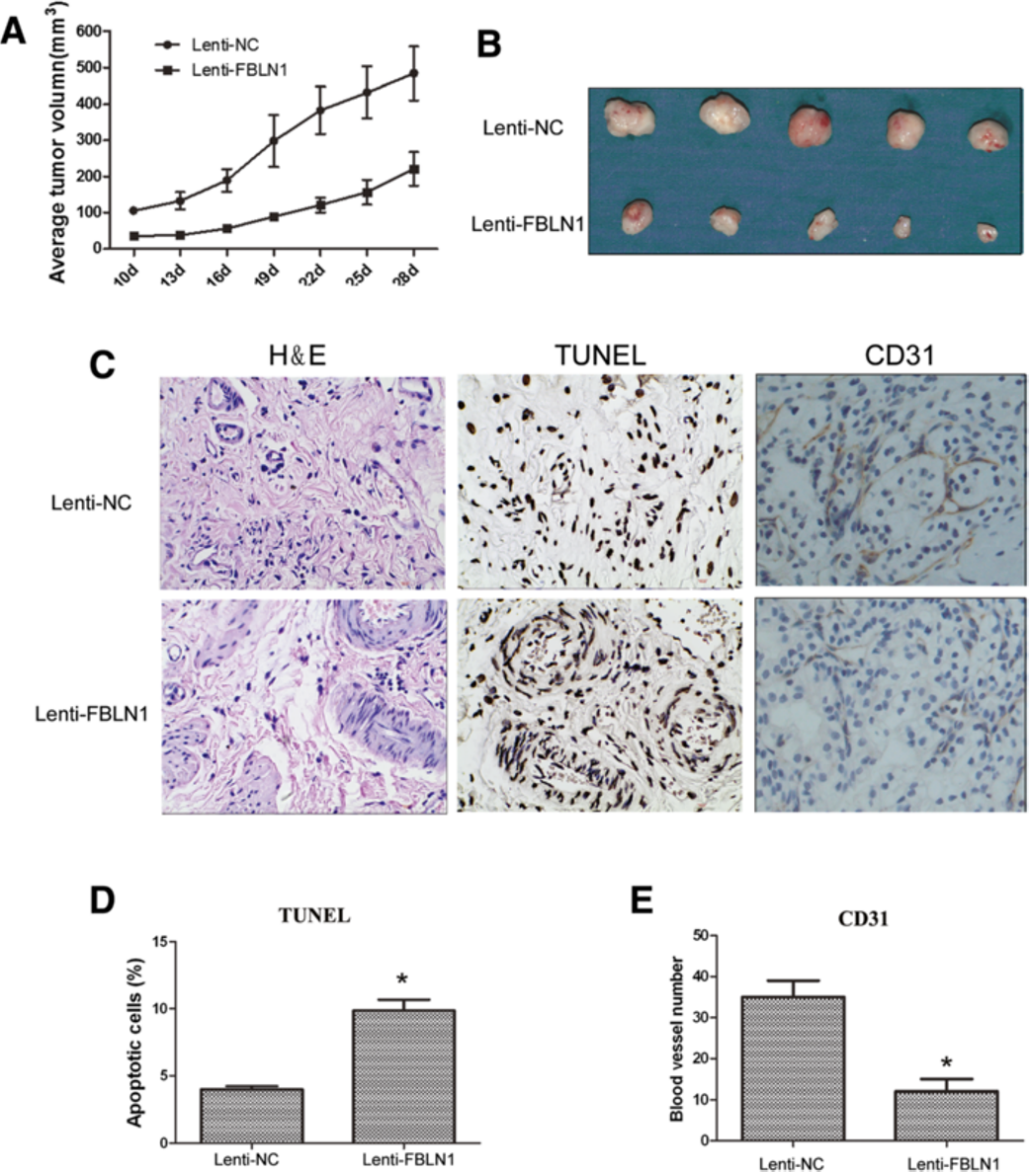
**Fibulin-1 suppressed bladder cancer** ***in vivo.*** Five mice were used per group conducted by 5637 cells transduced to express blank vehicle (Lenti-NC) or fibulin-1 (Lenti-FBLN1). **A)** Tumor growth curves of xenograft inoculated in nude mice. Curves represent average tumor size of 5 mice per time point. **B)** Representative images showed the xenograft tumors. **C)** Representative tumor sections of xenograft tumors derived from 5637 cells transduced to express Lenti-NC (a–c) and Lenti-FBLN1 (d–f). (a) and (d) show H&E stained images; (b) and (e), TUNEL of the xenograft tumors, white arrows showed the positive plots; (c) and (f), immunohistochemistry for CD31 (in dark brown color). **D)** Quantitative analyses of TUNEL are shown as mean ± SD of five randomly selected microscope fields per slice of mice in the two groups. **E)** The mean ± SD of blood vessel number of five randomly selected microscope fields per slice of mice in the two groups. Asterisk indicates p < 0.05.

We assessed FBLN1 tumor suppressor activity also *in vivo.* As shown in Figure 5A and B, the latency of tumor growth in mice with fibulin-1 expressing 5637 cancer line was significantly longer when compared to the control, and the average tumor volume in mice with fibulin-1 expressing group were significantly smaller than the control group.

The mice were sacrificed 28 days after tumor cell injections and tumors in each group were harvested and sectioned. Apoptosis in tumor samples was detected by TUNEL assay. Fibulin-1 induced apoptosis much more robustly when compared to the control (Figure 5C and D, P < 0.05). To further characterize the *in vivo* effects of fibulin-1 on angiogenesis, 5637 tumors were evaluated for blood vessel density. The tumors were labeled with CD31, an endothelial cell–specific marker. Immunohistochemistry results revealed that blood vessel quantification reduced drastically in the 5637 tumors over-expressing fibulin-1 (Figure 5C and E, P < 0.05), which indicated that fibulin-1 significantly inhibit bladder tumor angiogenesis.

## Discussion

Fibulin-1 is an extracellular matrix and plasma protein that has been implicated as playing a role in tumor progression [16, 20, 26–29]. Our data revealed the expression of fibulin-1 was significantly decreased or lost in bladder cancer tissues and cell lines. Using IHC analysis of 139 non-muscle invasive bladder cancer patient tissue samples, we found the expression of fibulin-1 in NMIBC was associated with tumor grade, indicated that loss of fibulin-1 expression may contribute to bladder cancer progression, these results accord with our previously observations of fibulin-5 in bladder cancer tissues [30]. Interestingly, the expression of fibulin-1 in cancer cell lines was a little different with tissue samples. 5637 cells, which derived from a grade 2 bladder transitional cell carcinoma had the lower fibulin-1 expression than J82 or T24 cells which originated from high grade, invasive human bladder cancer. Implying that the expression of fibulin-1 in muscle-invasive bladder cancer tissues might be different.

Epigenetic alterations such as promoter hypermethylation can lead to the transcriptional silencing of tumor suppress genes, which is important for preventing cancer development. Fibulin-1 has been found epigenetically silenced in gastric cancer and hepatocellular carcinoma through promoter hypermethylation [19, 20]. Using methylation-specific PCR and quantitative sequencing, we found the promoter regions of fibulin-1 were generally methylated in bladder cancer cell lines (5637, HT1376, J82 and T24) and tissues. Remarkably, we found the transcription level of fibulin-1 in bladder cancer cells increased as expected after 5-AZA-dC treatment, but it was still well below that in bladder non-tumorgenetic cell line SV-HUC-1. This observation indicated that there might be some other mechanisms that regulated fibulin-1 expression. For example, fibulin-1 maturation and mislocalisation have been found in breast cancer [27], while in prostate cancer, fibulin-1 was also down-regulated but did not induce by 5-aza-2′-deoxycytidine [31].

Many studies have investigated molecular biomarkers for prediction of risk and recurrence of bladder cancer. To date, several molecular markers have been reported to be associated with bladder cancer recurrence [32–34]. Our results now identified fibulin-1 expression as associated with the recurrence-free survival of non-muscle invasive bladder cancer patients. Although the sample size in our study was relatively small and needed further ascertain, to our knowledge, this is the first study to provide evidence that fibulin-1 expression may play an important role in the prediction of NMIBC recurrence.

In the latter part of the study, we systematically investigated the tumor suppressing function of fibulin-1 in bladder cancer cells. As a novel ECM protein, fibulin-1 had been reported a multifunction protein in variable aspects of tumor cells, such as cell motility [35], cell proliferation [20], apoptosis and angiogenesis [36]. Our findings from *in vitro* and *in vivo* experiments proved that overexpressing fibulin-1 suppressed tumor growth, induced tumor cell apoptosis, decreased cell motility, and inhibited angiogenesis in bladder cancer cells. These tumor suppressing functions of FBLN1 corresponded with its loss of expression in bladder cancers. We conjectured that the methylation degree of FBLN1 promoter increased in the progression progress of bladder cancer, which resulted in the loss of fibulin-1 expression, so that enhanced the bladder tumor growth and ability of metastasis and angiogenesis.

## Conclusions

In conclusion, our study provides evidences that FBLN-1 functions as a novel candidate tumor-suppressor gene in bladder cancers and its down-regulation maybe due to the promoter hypermethylation. Restoration of fibulin-1 expression significantly inhibited bladder cancer cell growth, motility and angiogenesis, which are indicative features of tumor suppressor gene. The dysregulation of fibulin-1 is associated with NMIBC grade and recurrence. Further studies are needed to determine the potential usefulness of assessing FBLN1 in bladder cancer as a prognostic marker and candidate target for gene therapy.

## Electronic supplementary material

Additional file 1: Table S1: Primer sets sequences used in this study. (DOC 34 KB)
