# Supplementary material for: Fibulin-1 is epigenetically down-regulated and related with bladder cancer recurrence
Source: BMC Cancer. 2014 Sep 18;14:677. doi: 10.1186/1471-2407-14-677 (PMC4180143; doi:10.1186/1471-2407-14-677)
Supplement: Supplementary file 1 — Additional file 1: Table S1: Primer sets sequences used in this study. (DOC 34 KB) [file 12885_2014_4867_MOESM1_ESM.doc]

Additional file 1: Table S1. Primer sets sequences used in this study.

| Primer | Sequence (5’-3’) | Tm |
| --- | --- | --- |
| Fibulin-1 | F: TGC GAA TGC AAG ACG G | 60℃ |
|  | R: CGT AGA CGT TGG CAC A |  |
| MSP-M | F: GTT TTT GTT TTT GAG GGT AGA GTC | 58℃ |
|  | R: AAA AAC ACT AAA ACA AAC CTT ACC G |  |
| MSP-U | F: GTT TTT GTT TTT GAG GGT AGA GTT G | 58℃ |
|  | R: AAA ACA CTA AAA CAA ACC TTA CCA C |  |
| Sequencing | F: GAG TGT TTT TTT GTG GTT GAT T | 50℃ |
|  | R: TTT GAT TTA AGT TTG TGA GA |  |
| GAPDH | F: GCA GGC GTC GGA GGG CCC CCT C  R: GGG ACT GAG TGT GGC AGG GAC TCC | 60℃ |
